# Supplementary figures and images for: On the agreement between bibliometrics and peer review: Evidence from the Italian research assessment exercises
Source: PLoS One. 2020 Nov 18;15(11):e0242520. doi: 10.1371/journal.pone.0242520 (PMC7673579; doi:10.1371/journal.pone.0242520)

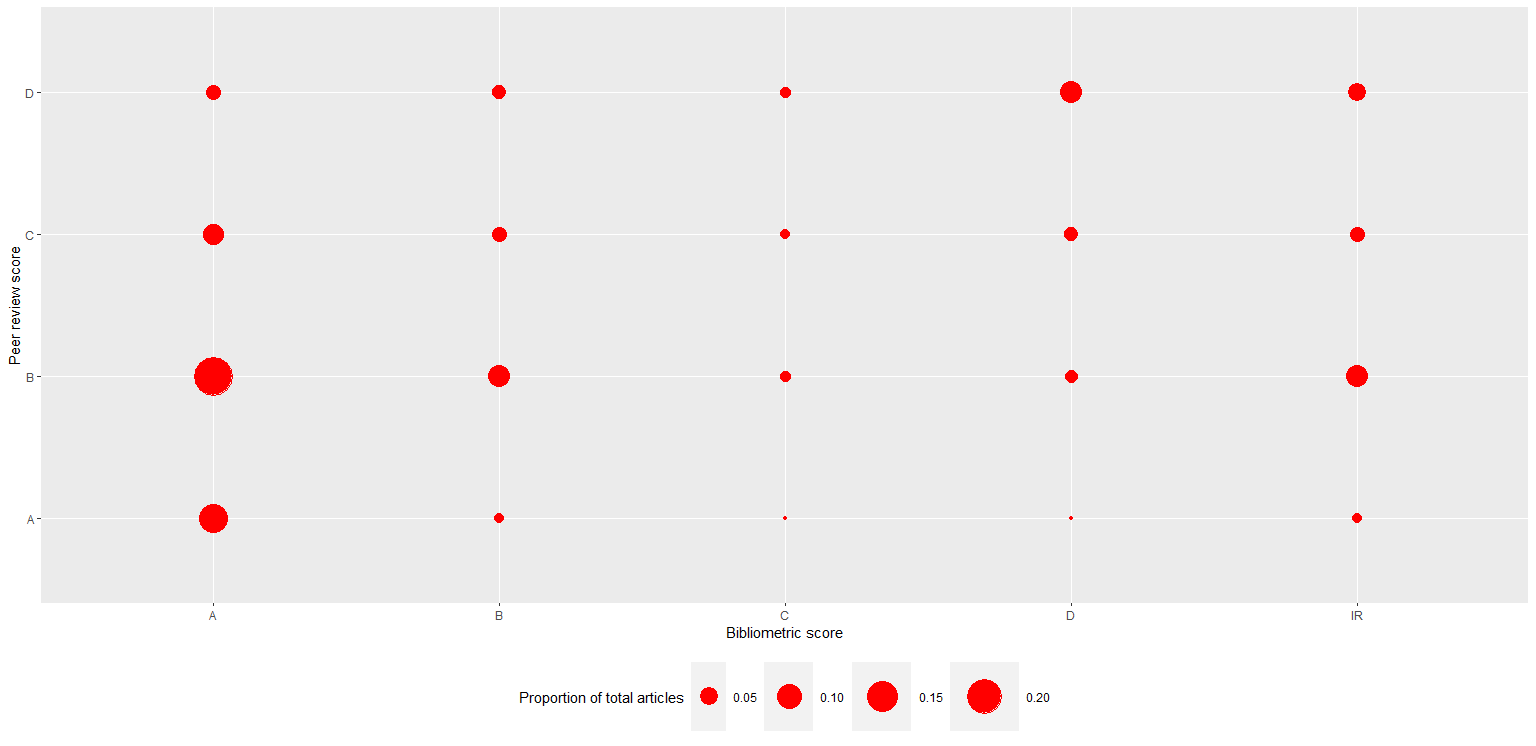

Supplement: S1 Fig — Count overlapping points (proportion). All research areas. (TIF) [file pone.0242520.s001.tif]

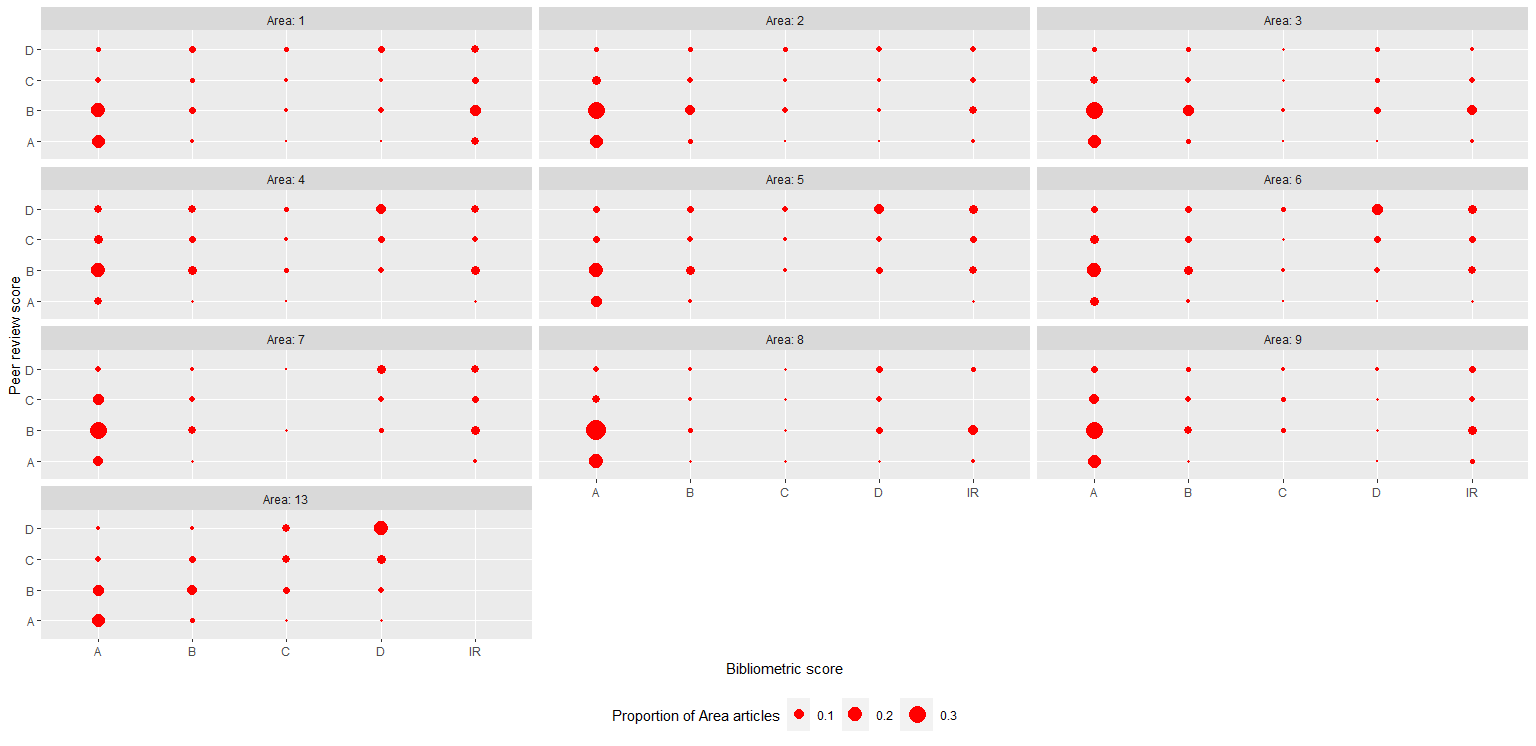

Supplement: S2 Fig — Count overlapping points (proportion). Separate plot for each research area. (TIF) [file pone.0242520.s002.tif]

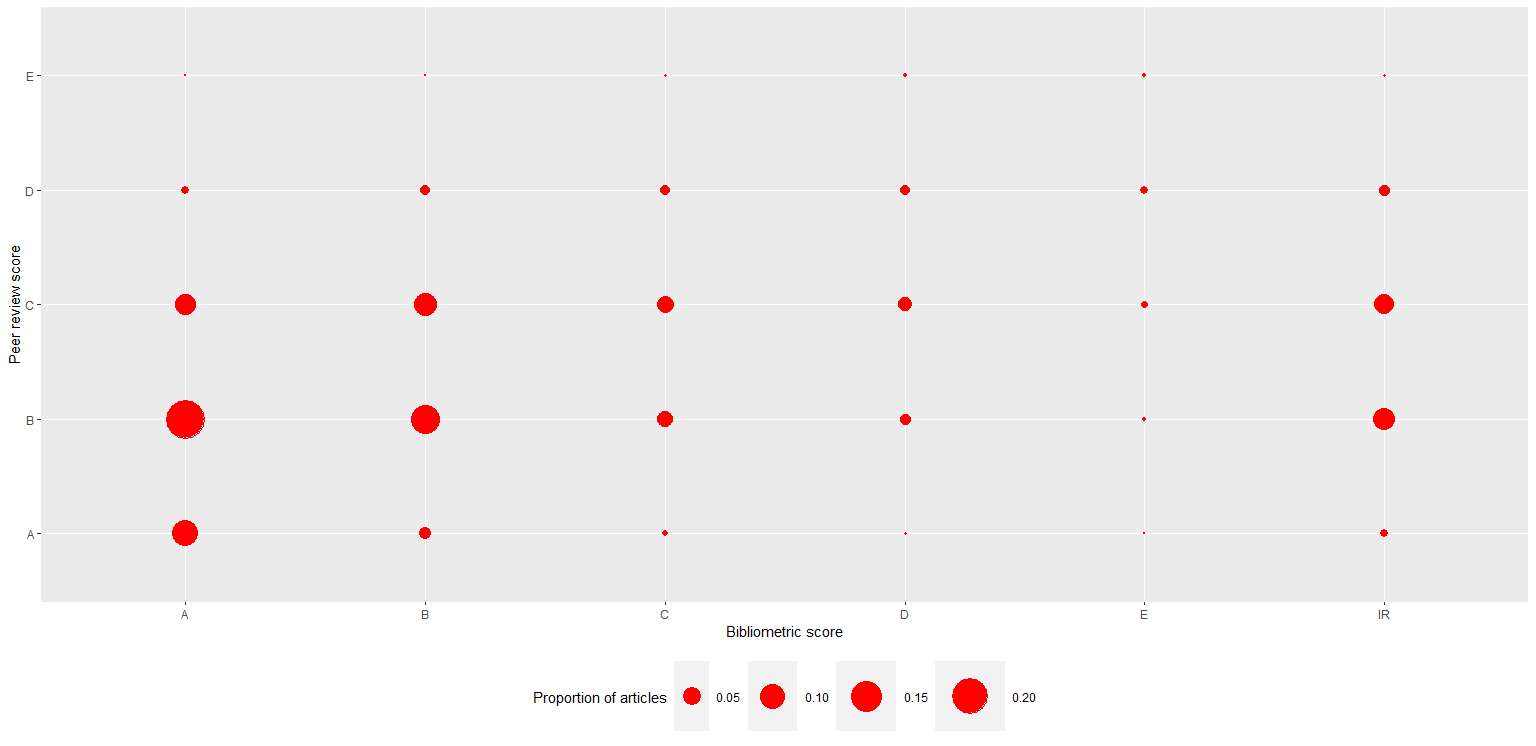

Supplement: S3 Fig — Count overlapping points (proportion). All research areas. (TIF) [file pone.0242520.s003.tif]

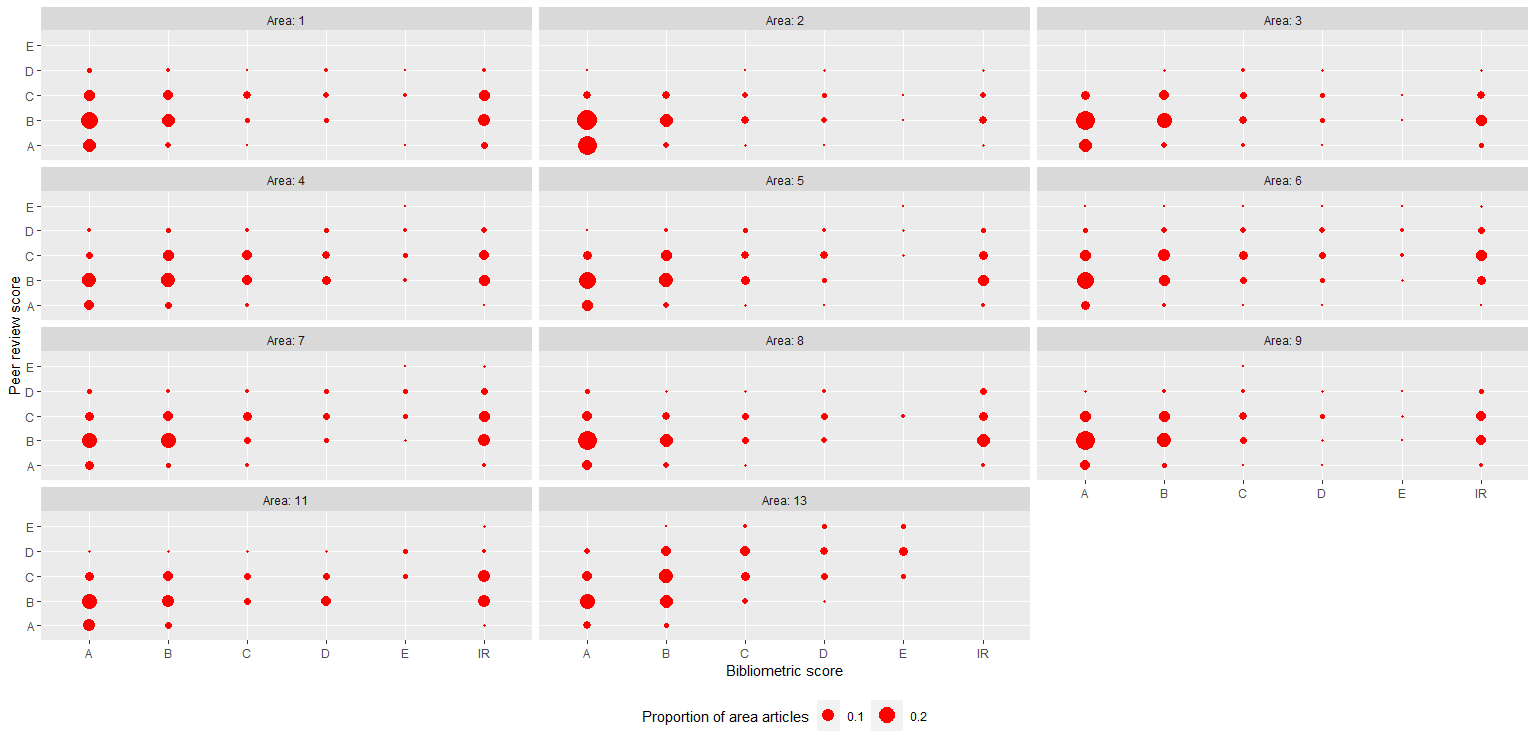

Supplement: S4 Fig — Count overlapping points (proportion). Separate plot for each research area. (TIF) [file pone.0242520.s004.tif]

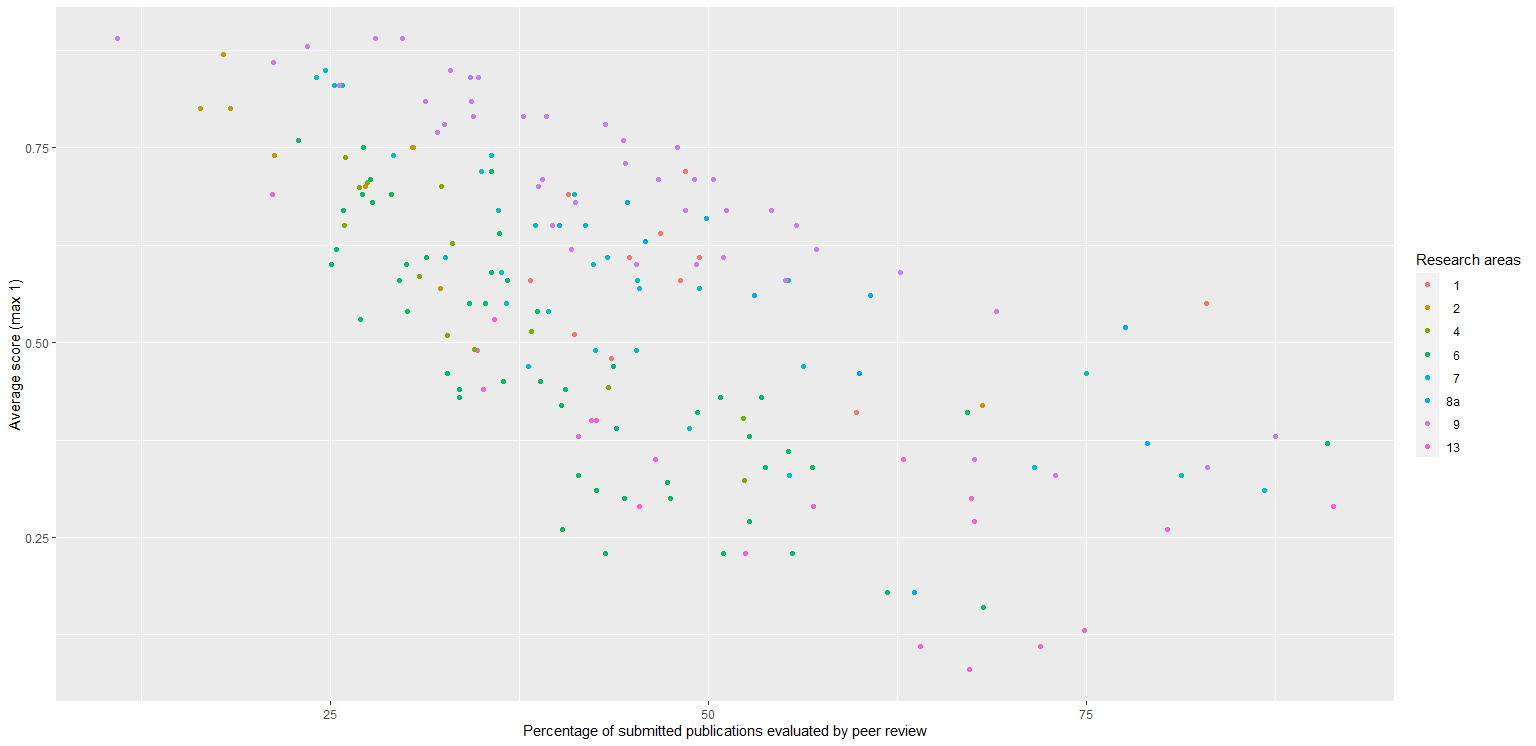

Supplement: S5 Fig — All areas. (TIF) [file pone.0242520.s005.tif]

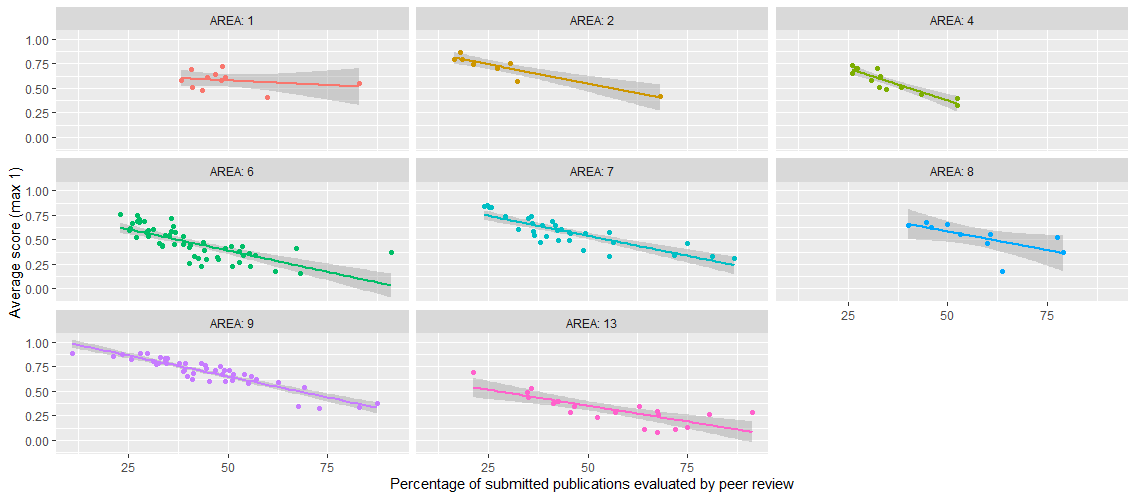

Supplement: S6 Fig — VQR1. (TIF) [file pone.0242520.s006.tif]

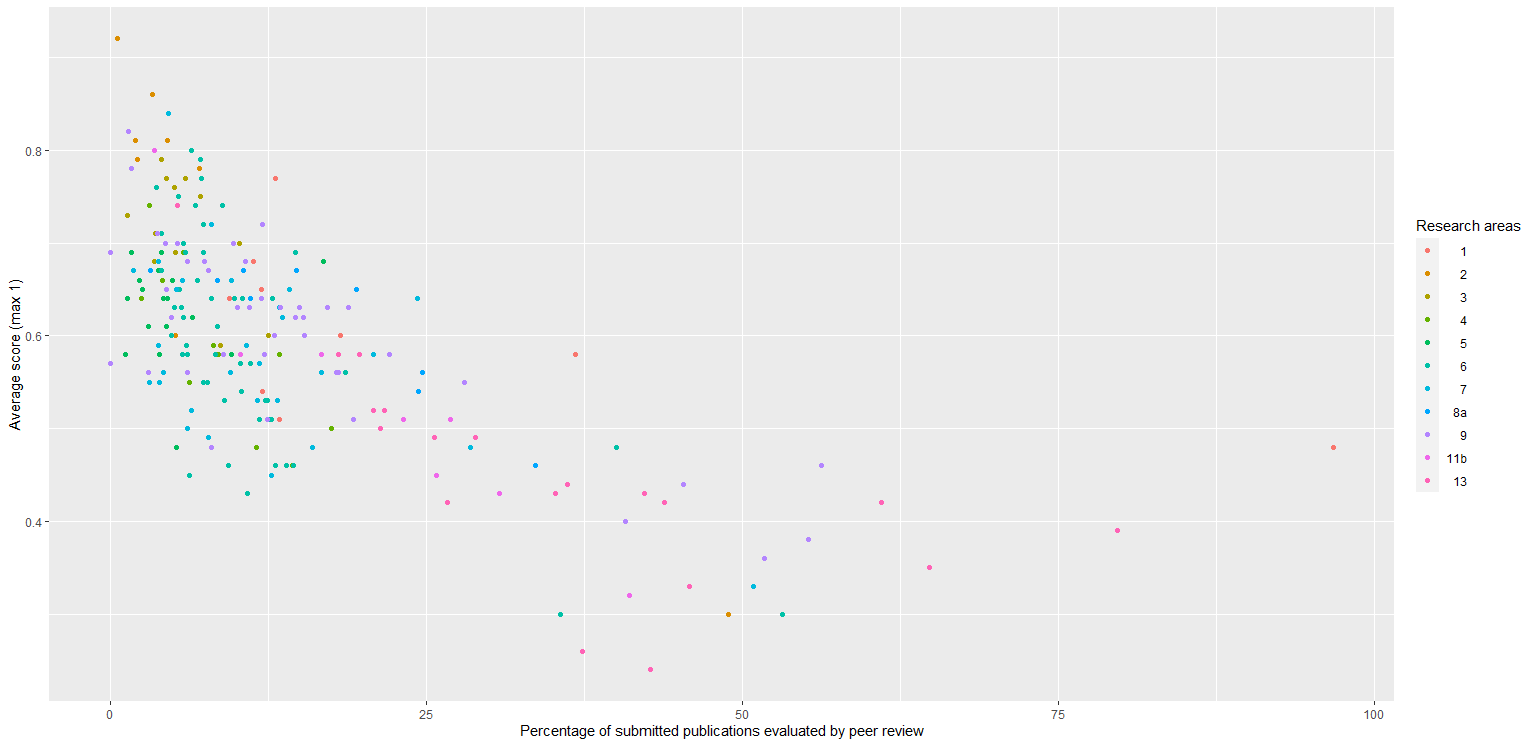

Supplement: S7 Fig — All areas. (TIF) [file pone.0242520.s007.tif]

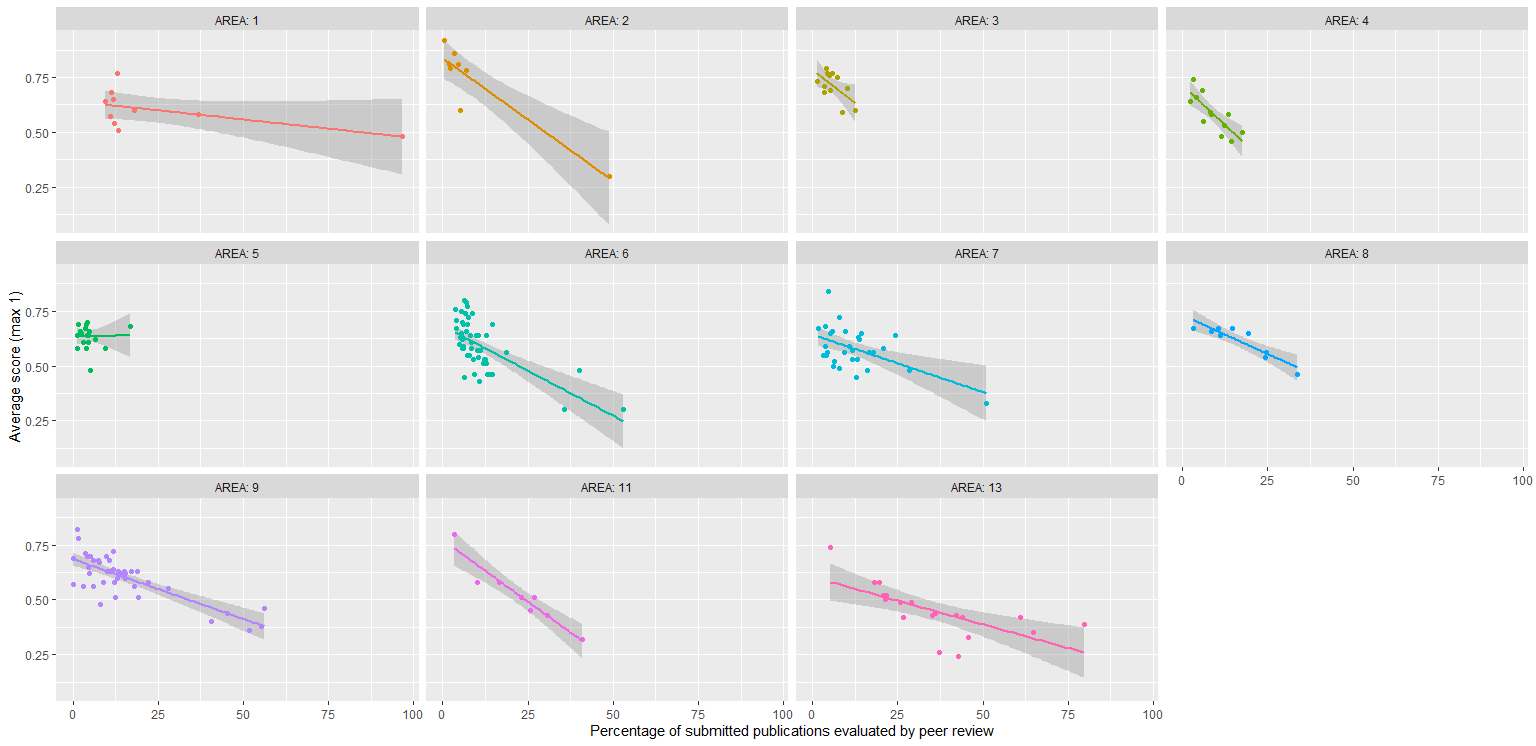

Supplement: S8 Fig — VQR2. (TIF) [file pone.0242520.s008.tif]
