# Supplementary material for: On the agreement between bibliometrics and peer review: Evidence from the Italian research assessment exercises
Source: PLoS One. 2020 Nov 18;15(11):e0242520. doi: 10.1371/journal.pone.0242520 (PMC7673579; doi:10.1371/journal.pone.0242520)
Supplement: S1 Table — 4 Tables. (PDF) [file pone.0242520.s011.pdf]

On the agreement between bibliometrics and peer review: evidence from the Italian research assessment exercises. Supplementary tables.

Alberto Baccini, Lucio Barabesi, Giuseppe De Nicolao

The following four tables synthesize Cohen's kappa estimates obtained by adopting the linear weights described in Section 6 of the paper.

Table A.1: Cohen's kappa estimates with linear weights (percent) for EXP1 (95% confidence level intervals in parenthesis), bibliometric vs peer rating.

Area	ANVUR[a]	$\widehat{\kappa}_w^{(1)}$	$\widehat{\kappa}_w^{(2)}$	$\widehat{\kappa}_w^{(3)}$
1	31.76(24.00,40.00)	31.76(26.03,37.48)	33.28(27.53,39.02)	15.96(12.90,19.02)
2	23.02(19.00,27.00)	23.02(19.59,26.45)	26.16(22.85,29.47)	18.06(15.68,20.43)
3	22.46(17.00,28.00)	22.46(18.23,26.69)	23.65(19.44,27.85)	15.33(12.43,18.23)
4	27.76(20.00,36.00)	27.26(21.52,32.99)	27.54(21.79,33.28)	18.72(14.60,22.84)
5	32.87(28.00,38.00)	32.87(29.31,36.43)	34.59(31.14,38.05)	22.83(20.38,25.29)
6	30.24(26.00,34.00)	30.30(27.43,33.18)	31.25(28.43,34.07)	20.74(18.74,22.73)
7	27.76(21.00,34.00)	27.76(22.42,33.11)	29.76(24.64,34.89)	19.20(15.66,22.74)
8a	19.94(10.00,30.00)	19.94(11.62,28.26)	20.34(12.01,28.66)	15.12(8.68,21.55)
9	16.15(12.00,20.00)	16.15(12.93,19.37)	19.78(16.36,23.20)	12.36(10.20,14.51)
13	54.00(46.00,62.00)	54.41(49.89,58.92)	54.41(49.89,58.92)	54.41(49.89,58.92)
All	31.52(30.00,33.00)	31.23(29.91,32.55)	32.60(31.30,33.89)	21.89(20.95,22.82)

[a] Source: ANVUR (2013, Appendix B). Reproduced in Ancaiani et al. (2015).

Table A.2: Cohen's kappa estimates with linear weights (percent) for EXP2 (95% confidence level intervals in parenthesis), bibliometric vs peer rating.

Area	ANVUR[a]	$\widehat{\kappa}_w^{(1)}$	$\widehat{\kappa}_w^{(2)}$	$\widehat{\kappa}_w^{(3)}$
1	21.30(15.00,27.60)	21.28(15.25,27.31)	22.64(16.57,28.72)	14.61(11.56,17.66)
2	26.40(22.40,30.50)	26.41(22.55,30.27)	28.59(24.80,32.38)	21.56(18.75,24.38)
3	19.40(14.30,24.60)	19.44(14.57,24.31)	20.79(15.97,25.62)	13.08(10.20,15.95)
4	23.70(16.50,30.90)	23.67(16.87,30.47)	24.25(17.57,30.94)	14.97(10.95,18.99)
5	24.00(19.70,28.30)	24.03(19.95,28.11)	24.96(20.93,28.98)	20.03(17.91,22.15)
6	22.70(19.30,26.00)	22.68(19.49,25.86)	24.38(21.25,27.51)	21.52(20.07,22.97)
7	26.50(21.00,32.10)	26.54(21.32,31.77)	28.43(23.35,33.51)	14.95(12.17,17.72)
8b	17.10(8.80,25.40)	17.08(9.135,25.03)	20.22(12.48,27.96)	10.71(6.76,14.66)
9	16.80(12.80,20.80)	16.78(12.95,20.61)	19.47(15.71,23.23)	19.00(17.11,20.89)
11b	23.50(13.50,33.40)	23.46(14.08,32.84)	25.02(15.87,34.17)	13.96(9.13,18.79)
13	30.30(25.70,34.80)	30.27(25.91,34.63)	30.27(25.91,34.63)	31.31(27.46,35.17)
All	25.80(24.30,27.40)	25.93(24.48,27.37)	27.13(25.71,28.54)	20.69(19.89,21.49)

[a] Source: ANVUR (2017, Appendix B, Table B.7).

Table A.3: Cohen's kappa coefficient estimates with linear weights (percent) for EXP1 (95% confidence level intervals in parenthesis), P1 vs P2 ratings.

Area	ANVUR[a]	$\widehat{\kappa}_w^{(1)}$	$\widehat{\kappa}_w^{(1)}$ (DBR)[b]	$\widehat{\kappa}_w^{(1)}$ (IR)[c]
1	35.95(27.00,45.00)	33.55(28.10,39.00)	35.95(26.75,45.15)	27.65(17.45,37.85)
2	23.32(18.00,29.00)	23.91(20.16,27.66)	23.37(18.46,28.28)	17.99(8.584,27.39)
3	25.01(19.00,31.00)	22.19(17.63,26.74)	25.01(19.29,30.72)	2.718(-7.02,12.45)
4	25.00(15.00,35.00)	23.03(16.56,29.49)	25.00(16.56,33.45)	12.69(-2.37,27.76)
5	27.50(22.00,33.00)	25.52(21.66,29.38)	27.50(22.33,32.68)	11.23(2.548,19.92)
6	24.60(20.00,29.00)	22.88(19.76,26.00)	24.66(20.50,28.82)	11.48(4.19,18.76)
7	15.70(7.00,24.00)	16.54(10.74,22.34)	15.70(7.57,23.83)	16.44(3.67,29.22)
8a	20.29(7.00,33.00)	19.88(10.26,29.50)	20.29(8.45,32.13)	16.92(-11.70,45.55)
9	19.35(13.00,25.00)	20.60(16.40,24.80)	19.35(13.50,25.21)	22.57(12.88,32.27)
13	40.00(32.00,48.00)	39.50(34.36,44.65)	39.50(34.36,44.65)	-
All	28.53(26.00,31.00)	27.15(25.72,28.58)	28.30(26.42,30.17)	18.95(15.54,22.35)

[a] Source: ANVUR (2013, Appendix B). Reproduced in Ancaiani et al. (2015).
[b] Weighted Cohen's kappa for the sets of articles with a definite bibliometric rating (DBR).
[c] Weighted Cohen's kappa for the sets of articles without a definite bibliometric rating and submitted to informed peer review (IPR).

Table A.4: Cohen's kappa coefficient estimates with linear weights (percent) for EXP2 (95% confidence level intervals in parenthesis), P1 vs P2 ratings.

Area	ANVUR[a]	$\widehat{\kappa}_w^{(1)}$	$\widehat{\kappa}_w^{(1)}$ (DBR)[b]	$\widehat{\kappa}_w^{(1)}$ (IR)[c]
1	19.90(12.60,27.10)	23.52(17.37,29.67)	19.86(10.92,28.81)	35.00(21.63,48.38)
2	19.40(14.50,24.30)	21.00(16.64,25.36)	19.4(14.17,24.63)	19.92(6.79,33.04)
3	13.90(7.80,20.00)	14.59(9.31,19.86)	13.93(7.12,20.74)	15.30(2.97,27.63)
4	18.70(11.10,26.50)	18.20(11.68,24.72)	18.71(10.07,27.36)	11.28(-2.41,24.97)
5	19.20(14.30,24.10)	19.78(15.45,24.11)	19.17(13.40,24.95)	19.97(9.46,30.49)
6	18.70(14.60,22.70)	17.63(14.08,21.17)	18.66(13.87,23.46)	8.86(0.38,17.35)
7	19.30(13.20,25.84)	22.00(16.86,27.15)	19.28(11.39,27.17)	27.61(17.24,37.97)
8b	3.40(-6.20,12.90)	8.47(0.17,16.78)	3.36(-9.25,15.98)	21.87(5.65,38.09)
9	15.00(9.80,20.20)	15.18(10.7,19.66)	15.00(8.85,21.16)	12.21(1.53,22.89)
11b	25.30(13.10,37.60)	26.03(15.55,36.51)	25.32(8.03,42.61)	22.96(1.19,44.72)
13	31.40(25.70,37.10)	31.38(25.99,36.76)	31.38(25.99,36.76)	-
All	23.20(21.40,25.00)	23.35(21.79,24.90)	23.36(21.35,25.36)	19.59(15.75,23.44)

[a] Source: ANVUR (2017, Appendix B, Tabella B.7).
[b] Weighted Cohen's kappa for the sets of articles with a definite bibliometric rating (DBR).
[c] Weighted Cohen's kappa for the sets of articles without a definite bibliometric rating and submitted to informed peer review (IPR).
