# Supplementary material for: On the agreement between bibliometrics and peer review: Evidence from the Italian research assessment exercises
Source: PLoS One. 2020 Nov 18;15(11):e0242520. doi: 10.1371/journal.pone.0242520 (PMC7673579; doi:10.1371/journal.pone.0242520)
Supplement: Attachment — Submitted filename: RefereeReply.pdf [file pone.0242520.s012.pdf]

Reply to Reviewer #1:

General comment. *This paper is an evaluation of the results of a previous experiment (EXP1/2) to test the approximate equivalence of bibliometric and peer review methods of evaluating the quality of departmental research. A governmental research agency (ANVUR) evaluated research quality across multiple universities and fields (The range of fields was very broad, including social sciences and humanities). They did this using an "evaluative mix" of bibliometric algorithmic ranking or the use of peer review for a large bolus of research output (including peer reviewed journal articles as well as other research outputs). Peer reviewed articles were bibliometrically evaluated, while books and other research outputs were evaluated via a peer review panel. If the algorithm did not return an internally consistent result (IR), for instance a high citation count but a low impact factor, the paper was peer reviewed instead. This seems poorly justified from the beginning. The agency then conducted an experiment (EXP1/2) with a sample from this bolus to test whether bibliometric and peer review evaluations were roughly equivalent, although it's not entirely clear if new panels were formed to peer review publications which were originally bibliometrically assessed. The sample was stratified across all topic areas, although it did not include any IR data, which the authors suggest may introduce a bias in the results. The authors now suggest to test the homogeneity of the stratified sample using a statistical method they have developed and their results have called into question the representativeness of the sample and therefore the conclusions of the original experiment, which was to equate bibliometric and peer review assessment. The original schema to evaluate research is very complex and the original experiment (EXP1/2) is also not completely described. For instance, its unclear if a new set of peer reviewers were employed in EXP1 to re-review outputs, so that two quality evaluations (one originally bibliometrics and one by IPR) for the exact same article can be compared to generate the Cohen's kappa agreement? Also, why did they conduct 2 expts (EXPT 1/2). Is there no difference? This paper would do well with a schematic or figure outlining how the ANVUR experiment was conducted.*

Reply. Dear referee, many thanks for your comments.

On the basis of a comment of referee #2 we have decided to re-design the whole paper. In addition, we had access to the raw individual data of the two experiments analyzed in the former manuscript. The data were solely disclosed by the Italian National agency for research evaluation in April 2019. Therefore, it was impossible for us to complete a revision in due time in such a way that

we provisionally decided to withdraw the submission. Moreover, the availability of data permitted to us also to enrich the analysis so that the revised version of the paper contains a more complete appraisal of the concordance between peer review and bibliometrics. The paper contains a complete reassessment of the results of the two experiments realized by the Italian governmental agency for research evaluation. As a consequence we have also introduced a more detailed exposition of the complexities of the original procedures. We have introduced a detailed explanation of the formation of the panels for peer reviews. We hope that the description of the procedure is now clearer than in the former draft. In order to draw evidence from these data we have defined the correct statistical setting of the experiments, and we have developed further original statistical tools. In particular, we have developed the design-based estimation of the Cohen's kappa coefficient and some further procedures for assessing the homogeneity of missing proportions between strata in a Bayesian framework. We agree with you that the the two experiments "seem poorly justified from the beginning". Notwithstanding, results of these experiments are published in peer reviewed journals, they are cited and considered as part of the received knowledge about research assessment. We hope that this new version of the paper is able to clarify the information that can be drawn from the experiments. We hope also that this new version of the paper contains results of general interest for scholars and policy makers interested in the design of research assessment exercises.

We realize that you have now to referee a rather different version of the original paper, but we hope that we have improved the presentation.

Comment 1. *Another confusing aspect of the original experiment is that, if peer review is to be avoided due to its subjective and potentially biased results, why then would the "Holy Grail" be to find a bibliometric process which yields identical results to it, as was seemingly the goal of EXP1. Also, it's known bibliometrics have limitations and biases inherent as well. Altogether, it's very hard to piece together the academic importance of the original EXPT1.*

Reply. The design of the Italian research assessment exercises is very complex. The decision to use both peer-review and bibliometrics for evaluating papers introduced many problems in the research assessments. This decision originated probably by the necessity to evaluate some fields where bibliometrics has a high coverage, and fields, notably human and social sciences, where the coverage of bibliometrics is poor.

We generally agree with your comment. So we have dropped reference to the Holy grail, by discussing more in details the question of interchangeability between peer-review and bibliometrics.

Comment 2. *In terms of the present study, some clarification is also needed. For instance, for Table 1 and 2 it seems from the text to suggest they are data from EXPT 1 and 2, but the Tables heading lists EXPT1 for both. Also, it is not completely clear why the invention of a statistical test is needed to show that data was removed from the sample in an inhomogeneous way. More justifi-*

cation should have been provided. Maybe I missed the point, but just in looking at the data in Table 2, from Area 1 - 22 % of the data were removed (removed 100 from original sample of 444) to make the sub sample while in Area 2 – 8 % of the data were removed (removed 82 from original sample of 1008). This alone seems to suggest they removed more data from some areas than others. More to the point, the very fact the original authors of EXP1 removed these points AT ALL does seem to suggest the potential for bias in the results, so its unclear what extra the statistical test adds to this argument.

Reply. Also in this case we completely agree with your comments. Two of the authors of the present paper (Baccini and De Nicolao) highlighted precisely this point in a letter to *Research Evaluation* (10.1093/reseval/rvx013). The same journal published a reply to the letter by pointing out that this simple fact is not enough. This is the reason we have done a lot of work for developing statistical tests.

Comment 3. *Overall, the authors should be commended for revealing the weaknesses of the previous work, which seems poorly designed and overreaching in its conclusions and may it seems have negative policy implications if taken on face value. Their suggestion that the sampling is not representative seems appropriate. That said, a bit more detail and justification should probably be provided to help the reader understand the complex context as well as why this statistical model is needed to call the previous research into question.*

Reply. As anticipated in our reply to the general comment, we hope that in this revised version the complex contest of the discussion is clearly exposed.

Comment 4. One minor comment, the authors mention "the article is organized as follows: in Section 2 we present the..." The authors should probably refer instead to the headings and sub-headings.

Reply. You are right. The structure of paper is changed and also the description of its organization.

Reply to Reviewer #2:

General comment. *The authors study a sample of publications used by the Italian agency of research evaluation (ANVUR) in several studies. In particular, they question whether the sample has equal proportions of "missing" items in different strata. They develop a statistical methodology for this specific situation. The authors find that the proportions are not equal in the different strata. Although I have some questions around the technical aspects, the results seem correct. Their conclusion that this is problematic for the studies that are undertaken using this sample, seems to be too strong. Let me elaborate on both points.*

Reply. Dear referee, your main comment (Comment 1 below) induced us to re-design the whole paper, since – in addition – we had access to the raw individual data of the two experiments analyzed in the former manuscript. The data were solely disclosed by the Italian National agency for research evaluation

in April 2019. Therefore, it was impossible for us to complete a revision in due time in such a way that we provisionally decided to withdraw the submission. Actually, the availability of data permitted to us also to enrich the analysis so that the revised version of the paper contains a more complete appraisal of the concordance between peer review and bibliometrics. We hope that this new version of the paper contains results of general interest for scholars and policy makers interested in the design of research assessment exercises. The paper contains a complete reassessment of the results of the two experiments realized by the Italian governmental agency for research evaluation. In order to draw evidence from these data we have defined the correct statistical setting of the experiments, and we have developed further original statistical tools. In particular, we have developed the design-based estimation of the Cohen's kappa coefficient and some further procedures for assessing the homogeneity of missing proportions between strata in a Bayesian framework. We realize that you have now to referee a rather different version of the original paper, but we hope that we have improved the presentation.

Comment 1. *First, let us discuss the technical aspect of the developed methodology. The authors propose that M_l is the number of missing items in stratum l, which is assumed to be a random variable distributed as M_l ∼ Binomial(N_l, pi_l). However, the number of missing items in each stratum is a given observable, not a random variable. I therefore do not understand the use of this random variable. The authors then proceed to provide the probability of drawing X_l=x "missing" items from M_l=m "missing" items in the population, when drawing in total n_l items from N_l total items in the population, which indeed follows a hypergeometrical distribution. They then proceed to argue that when M_l is marginalized out, we arrive at the binomial distribution, and that X_l ∼ Binomial(n_l, pi_l). Of course the derivation is correct, but I would argue that M_l should not be marginalized out. The observed value of M_l is simply given. If they do marginalize out M_l, they are effectively testing for equal proportions in the population, not whether the sample is a "representative" reflection of the population.*

Reply. Thank you for this comment. You are right, we had not well understood the nature of data until we had access to the whole individual dataset. Actually, true missing data solely arise in the second experiment (EXP2). The two experiments are now adequately described in details in the new Sections 3 and 4 and the difference between papers classified as IR and true missing papers is fully described. Hence, our findings for testing the homogeneity of missing data between strata are now introduced in Section 7 and correctly applied to the data arising in EXP2. In such a case the proposed inferential setting is appropriate. Indeed, as accurately explained in Section 3, in EXP2 some papers did not receive a peer-review evaluation since some reviewers refused to do it. Hence, the size of missing items M_h in the h-th stratum is actually a random variable, since it is can be solely observed a posteriori to sampling. Please, note that we are forced to change some symbols in Section 7 in order to avoid overlapping with the notations introduced in the new sections - in particular,

the symbol π_l is replaced by the symbol θ_l.

Comment 2. *The authors formulate the null hypothesis as pi_l = pi for all l, and the alternative hypothesis as pi_l != pi for at least one l. However, the likelihood function under the alternative hypothesis is not that there is at least one pi_l that is different from pi, but that all proportions are independent from each other. Could the authors please clarify this?*

Reply. We have not well understood this comment. Actually, under the alternative hypothesis the random variables $X_1, \ldots, X_L$ are independent – and this also happens under the null hypothesis – while each X_h is distributed according to the Binomial law with parameters n_h and θ_h. Thus, the joint probability function may be factorised as

$$p_{X_1,\ldots,X_L}(x_1, \ldots, x_L) = \prod_{h=1}^{L} p_{X_h}(x_h) = \prod_{h=1}^{L} \binom{n_h}{x_h} \theta_h^{x_h} (1-\theta_h)^{n_h - x_h} \mathbf{1}_{\{0,1,\ldots,n_h\}}(x_h) \ .$$

Therefore, for a given realization $x_1, \ldots, x_L$ of $X_1, \ldots, X_L$, the likelihood is given by

$$L_1(\theta_1, \ldots, \theta_L) \propto \prod_{h=1}^{L} \theta_h^{x_h} (1 - \theta_h)^{n_h - x_h} \mathbf{1}_{[0,1]^L}(\theta_1, \ldots, \theta_L) \ .$$

Comment 3. *The authors then argue that the Chi^2 test statistic should be used for testing the null hypothesis. This is perfectly fine, but I can't seem to follow the formula that they propose (the first Eq. after l. 205). Following the usual definition of Chi^2 as T = sum_l (O_l - E_l)^2/E_l, where O_l is the observed values and E_l the expected values (under the null hypothesis), I arrive at* T(y) = sum_l (n_l pi_hat_l - pi_hat)^2/n_l pi_hat = sum_l n_l(pi_hat_l - pi_hat)^2/pi_hat. *Perhaps I am misunderstanding something here, but could the authors please clarify this?*

Reply. Actually, the present setting is similar to the case of a $(L \times 2)$ contingency table. Indeed, for each stratum there are two classes (missing and non-missing data), in such a way that the χ^2 statistics is given by

$$R = \sum_{h=1}^{L} \left(\frac{(n_h\widehat{\theta}_h - n_h\widehat{\theta})^2}{n_h\widehat{\theta}} + \frac{(n_h(1-\widehat{\theta}_h) - n_h(1-\widehat{\theta}))^2}{n_h(1-\widehat{\theta})} \right) = \sum_{h=1}^{L} \frac{n_h(\widehat{\theta}_h - \widehat{\theta})^2}{\widehat{\theta}(1-\widehat{\theta})} \ .$$

If you prefer, we can introduce the previous details in the text.

Comment 4. *Finally, I don't quite understand why the authors developed this statistical test specifically for this purpose. Isn't the end result a relatively straightforward test of equal proportions, using a Chi^2 test statistic? Of course, the equation for the P-value that is provided by the authors (in the Eq. just*

before l. 206)is different, because it is based on the multivariate hypergeometric distribution instead of the normal distribution usually assumed to arrive at the Chiˆ2 distribution for the Chiˆ2 test statistic. But the normal distribution is a reasonable approximation of the multivariate hypergeometric distribution (under some conditions), and so the difference with their calculation of the P value and the result from the Chiˆ2 distribution should not be too large. Could the authors please elaborate on this point?

Reply. We have adopted the present framework since the usual large-sample results for the distribution of the χ^2 test statistic for proportion homogeneity are precluded. Indeed, it is not possible to assume that the sample size n approaches infinity, since it is necessarily bounded by the population size N. In order to obtain the distribution of the test statistic, the Háyek-type Central Limit Theorem (see Hájek, J., 1960, Limiting distributions in simple random sampling from a finite population, Publications of the Mathematical Institute of Hungarian Academy of Sciences 5, 361-374, and subsequent references) should be adopted by assuming a sequence of populations of increasing sizes, in such a way that suitable conditions on each stratum size and sample size are fulfilled. However, even if the technical drawbacks might be by-passed, in any case rather heavy (and non-verifiable) conditions have to be assumed for achieving the large-sample distribution of the test statistic. In contrast, by adopting an approach based on conditional testing (Lehmann and Romano, 2005, Chapter 10) as provided in Section 7, exact inference may be obtained without assuming unnecessary conditions. In addition, in the same Section, we have also introduced a procedure for assessing missing probability homogeneity under the Bayesian paradigm.

Comment 5. *What the authors then test is indeed whether the samples have identical proportion of "missing" items. However, any difference does not arise from the sampling, but rather, from the fact that the populations have different proportions of "missing" items. So, the sample may still be a "representative" sample of the population, it is just that the population has different proportions of "missing" items.*

Reply. See our reply to Comment 1. We hope that the issue should be clear in the new version of the paper.

Comment 6. *This brings me to my point about the fact that the authors seem to draw too strong conclusions. The authors argue that the sample is flawed, and that no conclusions can be drawn from the sample. However, the sample is not flawed, and may still be "representative" of the population, but the population simply has different proportions of "missing" items. I don't see how this is problematic for the sample. Moreover, the "missing" items are in a sense a bit peculiar. An article is "missing" if an article is classified as "IR" because there is some disagreement between the journal impact and the article citation impact (the one is high where the other is low or vice-versa). The authors explain this well. The articles that are classified as "IR" will then be sent out for review, and will not be evaluated on the basis of the metrics.*

Therefore, the analysis of the agreement between peer review and the bibliometric outcome by definition cannot be done in that case. So, I don't understand the problem of the "missing" items concerning the agreement between peer review and metrics: those "missing" items are simply never evaluated using metrics. What if ANVUR would simply provide the results also for the IR articles, and show the peer review results for those articles? (It seems that ANVUR should be able to provide these results.) Would the unequal proportion of missing items then still be problematic for this analysis? If so, the authors should argue why.

Reply. See our reply to Comment 1. We hope that the issue should be clear in the new version of the paper.

Comment 7. *The authors do have a fair point when they say that peer review outcomes are generally lower than bibliometric outcomes, and this is also acknowledged in publications by ANVUR, if I understand correctly. Indeed, if more items are evaluated by peer review, this may then decrease the overall evaluation result. Hence, universities that have relatively more items evaluated by peer review may have lower evaluation results. But this has nothing to do with the sample, but with the unequal proportions of "missing" items in the population. Perhaps it has even more to do with unequal proportions of "missing" items for different universities than unequal proportions for different fields.*

Reply. We agree with your comment. We have introduced a short discussion of this point in the paper and we have added available evidences in the supplementary materials (S5 Fig.png, S6 Fig.png. S7 Fig.png, S8 Fig.png).

Comment 8. *In conclusion, I would recommend that the authors make two revisions. The authors should clarify that they are in fact testing the homogeneity of "missing" items in the population (using the sample). Secondly, the authors should make clear how their finding of unequal proportions of "missing" items would affect the analysis of the agreement of peer review and metrics. Finally, I think it is not correct to simply denounce citations to any literature produced by ANVUR, and the authors should revise their phrasing.*

Reply. We hope that we have appropriately addressed to your comments. In particular in revised version we have smoothed the whole tone of the paper, and we have more precisely refined the conclusions that can be drawn from the results of our work.

Minor comments.

Reply. We have corrected all the typos and missprints.

Details:

- Perhaps better to use "definitive" proof instead of "definite" proof?
Done.

- l. 17. "Others RADs" should be "Other RADs"

The sentence has been deleted.

- l. 39. "For all" should presumable be "above all", or "in particular" or "especially"

Done.

- l. 44. "not-zero" should be "non-zero" presumably.

Done.

- l. 74. "were" should be "was" (the subject is "aim", not "VQR1 and VQR2").

The sentence has been deleted.

- l. 199. "unit may be missed", perhaps better to use "item" or "article" instead of "unit"?

The sentence has been deleted.

*- l. 119. "one of the member*s*" instead of "member".*

The sentence has been deleted.

- l. 123. Probably the authors want to cite as Abramo and D'Angelo (2016, 2017) and Franceschini and Maisano (2017).

Corrected.

- Table 2, should refer to EXP2.

Done.

- I don't really understand the usage of the indicator function for all probability definition. If the domain is clear (which it usually is from context), then this indicator function is unnecessarily complicated.

We would like to maintain the usage of the indicator function in the formulas. However, if you prefer, we will try to eliminate the indicator function where it is possible.

- l. 200. "the size of missing units", I assume this should be the "number of missing units", or better yet, "number of missing items". At least I don't know what else the size should be.

Corrected.

- l. 267. The authors mention that the null hypothesis should also be rejected for the sub-areas, but this is only the case for some areas.

Corrected.
